# Navigating Uncertainty: Experiences of Older Adults in Wuhan during the 76-Day COVID-19 Lockdown

**DOI:** 10.3390/healthcare11222970

**Published:** 2023-11-16

**Authors:** Jianing Tang, Tangsheng Wang, Jessica Cottrell, Fanli Jia

**Affiliations:** 1 School of Foreign Languages and Literature, Wuhan University, Wuhan 430072, China; tangjianing@whu.edu.cn; 2 School of Marxism, Wuhan University of Technology, Wuhan 430070, China; wangtangsheng@whut.edu.cn; 3 Department of Biology, Seton Hall University, South Orange, NJ 07079, USA; jessica.cottrell@shu.edu; 4 Department of Psychology, Seton Hall University, South Orange, NJ 07079, USA

**Keywords:** COVID-19, social support, behavioral adaptation, elderly

## Abstract

The COVID-19 pandemic continues to affect the world. Wuhan, the epicenter of the outbreak, underwent a 76-day lockdown. Research has indicated that the lockdown negatively impacted the quality of life of older individuals, but little is known about their specific experiences during the confinement period. Qualitative interviews were conducted with 20 elderly residents of Wuhan, aged 65 to 85, who experienced mandatory isolation throughout the pandemic. The interviews centered around three stages of experiences: the Early Lockdown stage (the first week of lockdown after the government implemented the lockdown policy in January 2020), Infection During Lockdown stage (from February to April 2020 when participants were affected by the lockdown), and the Post-Lockdown stage (after April 2020 when the government lifted the lockdown policy). We found that older adults experienced different core themes during each lockdown stage. In the Early Lockdown stage, they felt nervousness and fear while searching for information. During the Lockdown and Infection Stage, they relied on reciprocal support and adjusted to new lifestyles. In the Post-Lockdown stage, they expressed cautions, trust, and gratitude. The finding highlights the evolving emotions and coping strategies of older adults throughout the lockdown phases. This study has yielded valuable insights into the adaptations of behavior and the importance of social interactions, specifically emphasizing the significance of healthcare among the elderly population.

## 1. Introduction

The Severe Acute Respiratory Syndrome Coronavirus 2 (SARS-CoV-2) was discovered in Wuhan, China, and resulted in a global pandemic. The World Health Organization officially named this new disease COVID-19 in February 2020. Consequently, the Epidemic Prevention and Control Headquarter of Wuhan issued a lockdown order. As part of the lockdown order, all buses, subways, ferries, and long-distance passenger transportation were suspended. Airport locations and railway stations were temporarily closed. As a result, citizens were unable to leave the city [1]. This lockdown order remained in place from 23 January 2020 to 8 April 2020, lasting a total of 76 days.

Social isolation resulting from the COVID-19 lockdown had a significant impact on elderly people’s emotional health and quality of life [2,3,4,5]. For elderly individuals living alone and with limited family, the social restriction associated with the COVID-19 pandemic may have contributed to a mental health crisis [6]. Social isolation involves limiting contact with family members, friends, neighbors (or home crowding with other family members), and home care services which can lead to difficulties in organizing or completing the delivery of essential items like groceries and medications [2,3,7,8]. Attitudinal barriers, such as feelings of unsafety in their neighborhood, further contribute to separation and loneliness among older individuals [9]. In many Chinese cities, the suspension of outdoor recreational activities (like visiting a park) and facilities like senior play centers and rehabilitation programs exacerbated the situation. Moreover, elderly caregivers who had children may have experienced increased caring burdens. Since children were required to stay at home during the lockdown, it was common that older adults were given decreased social contact and support [10]. The lack of meaningful activities and social relationships during the COVID-19 lockdown decreased the quality of life of older adults [4,5].

Unsurprisingly, increasing social support and contact has been recommended as an effective preventive strategy to maintain mental well-being and quality of life [2]. Several studies have been conducted to examine different forms of social support and social contacts available to older adults during the COVID-19 pandemic [10,11,12,13,14]. Formal social support services, such as adult day care, home-delivered meals, and transportation services have also been available to older adults during the pandemic. These services can help older adults maintain their independence and quality of life, even when they are unable to leave their homes. Steijvers et al. [11] examined ways in which family and friends can help older adults stay connected through informational, emotional, and practical support. Ottoni, Winters, and Sims-Gould [12] concluded that older adults relied on neighborhood connections and community outreach to stay connected during the pandemic. Nurain et al. [13] suggested that frequent contact with family members, friends, or neighbors via phone and social media helped mitigate loneliness and anxiety. 

It is important to note that many social services including homecare, home-delivered meals, and transportation services were restricted during the lockdown. This may have created significant fears and concerns among older adults [15,16,17], especially for those who relied heavily on these essential services for their daily living activities and social support. Moreover, not all older adults had access to the same forms of social support and contacts [11,18]. Some older adults were more isolated than others, and some had difficulty accessing the resources they need [19,20]. For example, older adults with transportation difficulties, chronic health conditions, or living alone were particularly restricted from obtaining social support services [19,20,21]. 

Recent studies have highlighted the potential of digital technologies in mitigating the negative impact of restricted social services on older adults [22,23]. When other traditional modes of communication were limited, digital technologies emerged as a valuable tool for older adults to stay connected with their community, family, and other emotional support networks [21]. However, a barrier to technological support is that older adults must be able to adapt (skill, access, and safety) to maintain social support. Older adults who were unable to use digital technology were more likely to experience social exclusion during the COVID-19 lockdown [22,23]. 

Although numerous studies have examined the impact of the pandemic on the lifestyles and well-being of older adults (see the review articles by Ahmadi et al. [24]; Derrer-Merk, [25]; Lebrasseur et al. [3]), there is limited information on their firsthand experiences and the strategies they employed to overcome lockdown challenges. We conducted a qualitative study through semi-structured interviews and analyzed the data using thematic analysis. We focused our research on understanding how older adults maintained their physical and psychological health, the composition of their social network, and identified interactions or elements that were significant to these older adults during the 76-day lockdown period. By interviewing older adults about how the lockdowns affected their daily lives, social connections, and mental/physical health, we can identify the specific ways they adjusted their mindsets and lifestyles to persevere through an exceedingly difficult time [23,24]. A qualitative study also allows for an in-depth exploration of diverse beliefs (e.g., majority and minority voices) to respond to various policies during the pandemic [26,27]. The diversity of behaviors and attitudes that emerged in response to the pandemic would go beyond surface-level survey assessments to reveal how individuals endured and found meaning in a significant disruption to their normal routine [25].

The current study aimed to explore the life experiences of older adults during the 76 days of the COVID-19 lockdown in Wuhan. To best assess psychological changes surrounding and throughout the lockdown period, our analysis focused on three stages: (1) Early Lockdown stage (the first week of lockdown after the government announced the lockdown policy), (2) Infection During Lockdown stage (from February to April 2020 when participants were affected by the lockdown), and (3) Post-Lockdown stage (late April 2020 after the government announced the lifting of the lockdown policy). Rather than examining individual differences or relationships among different variables via questionnaires, we emphasized the experiences and feelings of these older adults and provided a narrative perspective on what older adults may need from family, community, and society to maintain their health and well-being during and after the COVID-19 lockdown in Wuhan. This narrative understanding may offer a more comprehensive picture of the impact of the pandemic on older adults’ lives. Therefore, the goal of the study was to explore the life experiences of older adults during the 76-day lockdown period in Wuhan, China by considering their unique perspectives, challenges, and adaptive behaviors. 

## 2. Materials and Methods

### 2.1. Interview Guide Development

The purpose of the interview was to explore life experiences related to psychological, emotional, and physical well-being as well as social support during the 76 days of the COVID-19 lockdown. Semi-structured interviews were designed throughout the timeline of the lockdown. Stage 1: early lockdown; Sage 2: infection during lockdown period, and Stage 3: post-lockdown. 

We developed the interview guide according to outlines in previous qualitative studies on older adults’ healthcare [28,29,30]. Several questions were considered: How did the elderly gradually accept the information about the pandemic? What were their feelings? How did they perceive and understand the pandemic? How did the individuals react? How did they transition from emotional reactions to rational coping? What strategies did they use to effectively deal with and adapt to the lockdown of the pandemic? After conducting several rounds of discussions and preliminary testing with one older adult, we finalized the interview guide and decided to incorporate these questions into each stage of lockdown. We also avoided using complex or technical terminology that may be difficult for them to comprehend in terms of cognition and emotions. Overall, we asked the same ten questions to each participant with 24 specific prompts. All questions and prompts are listed in Supplementary S1. The COREQ (Consolidated criteria for Reporting Qualitative research) Checklist is included in Supplementary S2. 

### 2.2. Sampling Approach and Participants

Participants were recruited via two methods: personal connections and snowball sampling. The former personally reached out to individuals for their involvement in the study. The latter was achieved by placing advertisements in WeChat groups specific to retirees of various professions (e.g., teachers, blue-collar workers). These methods ensured a diverse group of participants broadly representing Wuhan’s older population regarding age, gender, marital status, living circumstances, academic background, residential district, and pre-retirement occupation. The inclusion criteria consisted of the following: (1) participants had to be over 65 years old (retirees), have resided in Wuhan during the lockdown, and had not been affected by the virus prior to the interview; and (2) participants needed to be capable of taking care of themselves in daily life, with only minor chronic diseases that did not significantly impact their overall lifestyle.

Twenty-one older adults between the ages of 65 and 85 (M_age_ = 68.65, SD = 4.30) participated in the study. All participants were residents of Wuhan and lived there during the lockdown period from 23 January to 8 April 2020. One participant was excluded from their interview because they did not meet the study’s inclusion criteria. Due to the sensitivity and high infection rate during the lockdown, we refrained from recruiting additional participants. Table 1 provides a description of their demographic characteristics. 

The first author obtained informed consent for each participant and conducted all the interviews online from 6 May to 20 June 2020. All interviews were conducted in Mandarin Chinese. All participants signed the consent form and agreed to participate in the study. They were allowed to withdraw from the study at any time and could refuse to answer any interview questions. The researchers remained neutral in collecting all the answers. The ethical and scientific values of the study were reviewed and approved by the Ethics Committee at Wuhan University.

### 2.3. Interview and Coding Procedures 

All participants were interviewed via telephone or video/audio conference call by the first author. Each interview took about 35–72 min. The interview guide was designed to achieve a content/thematic analysis that explored the participant’s personal experiences during the COVID-19 lockdown in Wuhan. 

All interview transcripts were imported into NVivo (Version 12), a qualitative data analysis software, to facilitate data management and analysis [31]. The coding process was conducted by three researchers who worked independently and coded the data according to predetermined stages (early, during, and after the lockdown). Each coder read the interviews carefully, identified the key concepts, and then grouped them together into themes. All coders engaged in regular discussions and reached an agreement on the coding decisions. The coding process was continued until no additional insights or themes were discovered from the interviews. This approach ensured that the analysis encompassed the various ranges of experiences and perspectives shared by the older adults.

## 3. Results

In this study, we explored psychological experiences among older adults in Wuhan during three stages of COVID-19. The first stage started from the lockdown of the city in February 2020, which has been named “Early Lockdown”. During this stage, the participants were aware of the seriousness of viruses and little information was shared from the official government. The second stage started from the infection point of the COVID-19 pandemic to the lift of the lockdown, and has been named “Infection During Lockdown”. The third stage from the lift of the lockdown in April 2020, to the time of the interview in early June 2020, has been named the “Post-Lockdown” stage. We generated 14 codes from the interview transcripts based on the seven key questions and prompts. After further refinement, we categorized these 14 codes into three main themes that correspond to different stages: Early Lockdown Stage, Infection During Lockdown Stage, and Post-Lockdown Stage. The 14 related codes during each stage of lockdown are summarized in Table 2. The participant’s code label (participant’s number, gender, and unanimous code) is included in the results. 

### 3.1. Stage One: Early Lockdown

#### 3.1.1. Stage One—Part A: Nervousness and Fear 

During the early phase of the lockdown, several factors contributed to the fragility of older people. These factors included the lack of general knowledge about the virus and uncertainty regarding the authenticity of the information received from television or the internet. Anxiety, fear, and nervousness were easily aroused among the elderly. Because there was a lack of accessible and clear data regarding the virus, older individuals became nervous. They were uncertain about who could be infected and the exact reasons why people were infected. Further, they were unsure how to avoid being infected, how many people were infected, so they were anxious and fearful about their own safety.

“At the very beginning, we thought this virus was very dangerous, and we didn’t know, it was very hidden, and we didn’t know how to avoid it. It was really worried in our heart, and we had never heard of it. This matter is going to be closed down, and I feel that this matter is very serious, just like a war, it feels like entering the air-raid shelter, you have to defend yourself and protect yourself. But it’s true how many people see this increase when you see it on TV? One day, how many were diagnosed? tens of thousands, how many in the country? Ah, I’m still quite nervous.”

(P20-F-CLS)—Participant#20, Female, Code CLS

“I think the epidemic situation of this disease is very serious. It was not like what was reported on the news. Preventable and controllable, at least add two zeros for the number of the infected individuals. I think this is the same as a rumor… If the city is closed on the 23rd, we would be nervous at this time. I think this is not a rare encounter in a century, that is, a rare encounter in a millennium, and there is no such thing as a lockdown.”

(P13-F-CMZ)

Older adults also described experiences from their early feelings that could be categorized into tension and fear, both of which contributed to the overarching sense of fear, anxiety, and nervousness. Several participants described their psychological reactions to hearing that relatives, neighbors, and acquaintances had been affected or lost their lives to COVID-19.

“A strong feeling is fear. There is a sense of fear, the invisible sense of fear, because of the incident of my sister and the death of myself.”

(P2-F-ZXY)

“The old lady next door to my son is nearly 89 years old. She is the same generation as us, almost the same age as us. The old lady is dead. I didn’t dare tell my son for a long time because I was afraid that they would be scared. I just told my son, if your neighbors are coming in or out, don’t open the door. If there is someone in the elevator, don’t go in. I don’t dare to tell them what happened to their home.”

(P13-F-CMZ)

Meanwhile, tension was used to describe external situations that older people encountered when shopping at a supermarket or purchasing medications at a pharmacy. They reported that the general environment of silence and awkwardness made them feel more worried and concerned. Two participants shared the following information:

“Later I also came back from that (sports) place... sometimes I felt so quiet when I saw it. It was almost eight o’clock. There was no one on the road, and there was no sound at all. At that time, it was in the invisible center. There is still a little bit of that kind of fear. A little bit of permeating feeling (now during the interview).”

(P14-M-CSS)

“I thought something was wrong, so I went to buy masks. I went to several shops and didn’t have them. Later, I went to a shop outside of our community, and a person whispered to me that masks might come in the evening. I kept going in the evening and finally came.”

(P6-F-TJ)

#### 3.1.2. Stage One—Part B: Searching for Support and Answers on Digital Media

There was a mixed use of media by older adults when seeking information. Through video calls, social media platforms, and online support groups, older adults were able to maintain social interactions, receive emotional support, and access information and resources.

However, media coverage of the pandemic during the lockdown period was filled with negative tones and reports from uncreditable sources, which naturally led to conflicting emotions and opinions. The influx of information was overwhelming for older adults, especially since many older adults are already suspicious of digital media. Several of the participants believed that digital information during the pandemic was too negative and felt distrustful and therefore avoided it.

“We have a Wechat group (Chinese social media) with my former colleagues, they always send lots of positive news information to me informing me how to focus on the pandemic, and how to take care of myself. That information is extremely positive. We are offering mutual support through the use of social media. Additionally, my son and daughter-in-law engaged in online shopping and shipped the necessary products from their city to Wuhan, as there was no direct online delivery available to Wuhan.”

(P3-F-WXJ)

“Initially, my focus was more on the epidemic. I used to frequently watch both domestic and international news on TV and the internet. However, after a while, I started feeling a bit exhausted and noticed a slight negativity creeping in. If I expose myself to more positive information, it becomes easier to comprehend and have a general understanding of it.

(P5-M-WTJ)

“We get up every morning to watch news on TV. Except for the news station of Channel 13 of the Chinese Central Channel, we mainly watch the Phoenix Information Channel, because we think news comes a little bit faster, and we generally don’t look at negative things, including WeChat’s negative ones. None of us reposted it.”

(P13-F-CMZ)

“Except for the topic of what we should do, I rarely read any of this news. I don’t want to read it. I don’t think it is useful. It only has a bad effect. There are also these official Italian and American news. How did all those people die? What I read was posted like this, I will delete it immediately, I will not read it. I think some are credible, and some are not necessarily credible, right. Why bother yourself. We just block some of these when we watch. I must see the things suggested in the community.”

(P9-F-QY)

### 3.2. Stage Two—Infection during Lockdown

#### 3.2.1. Stage Two—Part A: Adaptation and Social Support

During the lockdown period, older adults assessed the effectiveness of prevention and control measures while making necessary adjustments to their behavior. They gradually became confident in the capability of the government, community, and medical units to manage the lockdown. The participants in the second stage transitioned into a quietened state of mind during which they felt less nervous and fearful and were able to control their feelings. 

Several participants provided social support to others while they were receiving social support from the government, community, relatives, and friends. Interactions with others and receiving social support enhanced the mental well-being of those involved.

“I live alone in my home. From the time the community got to know my situation, they started to enlist me and provide various services to me, for example, giving me vegetables, rice, cooking oil, daily necessities and so on. Not only did they send everything to my apartment but also, they managed to deliver it to my front door. These are very detailed support.”

(P18-M-WGB)

“My next-door neighbors are always considering me, such as group shopping, they will ask me if I want a share of cake, they helped me a lot. So, I did believe that there are many downsides but also many positive sides of the pandemic i.e., it helped people feel closer with each other.”

(P20-F-CLS)

“My community staff even managed to shop for fresh fish and fruits. Those are rare supplies during the special time. It is extremely challenging for us to obtain fresh items during this difficult time; however, they not only managed to acquire them but also provide them free of charge.”

(P20-F-CLS)

#### 3.2.2. Stage Two—Part B: Providing Support to Others 

Participants were not only receiving social support from others, but were willing to devote themselves as well and act as social support providers, which in turn makes them feel positive.

“The staff at our apartment community have been invaluable during the epidemic. They continue to fulfill their duties, like taking care of us and buying medicine for residents when needed, despite the risks. They even went out of their way to help fix plumbing issues at some apartments. It’s worth noting that they’ve had to work much harder during this time but haven’t complained once... We were inspired to donate by a teacher in our community who started a donation initiative for these staff members. Many people, including us, followed their lead. We recognize how hard they work and the risks they take, so we wanted to show our appreciation.”

(P2-F-ZXY)

“We want to become a volunteer for our department community. We would like to conduct some basic works like disinfecting the community or distributing the groceries and so on. It is scared to go outside, but there should be someone to take the responsibility to do the job.”

(P2-F-ZXY)

“I was babysitting my grandson. I have a big family of three generations. I like contributing something for my family as far as I could.”

(P5-M-WTJ)

“I will take care of myself and limit the chances I ask for their help. I would like to contributor to them rather than be a burden.”

(P7-F-TAY)

#### 3.2.3. Stage Two—Part C: Adjusting to a New Lifestyle

Despite the challenges, older individuals gradually adjusted to a lifestyle that differed significantly from their previous routines. With restrictions preventing them from leaving their homes, many struggled to engage in physical exercise but found alternative ways to stay active indoors. They directed their attention towards culinary arts, cultivating their cooking skills and adopting healthier eating habits with a focus on a balanced diet. Establishing a stable daily schedule became essential for maintaining a structured lifestyle. Over time, these adaptive measures enabled older adults to cope with the situation and embrace their new reality at home.

“Eat more meat and high-protein things, otherwise you can’t get enough nutrition, and your resistance will not work. The most important thing is to strengthen and increase resistance. What we need each morning is a container of milk and an egg, and oranges every morning.”

(P13-F-CMZ)

“Try to eat a little bit better, with a balanced mix. My mom thinks he eats too much at night. I said you’re right. In any case, we should eat healthily to prevent problems such as thick blood.”

(P14-M-CSS)

“After the city was closed, I was alone at home, and I did the lessons taught by the (elderly university) teacher once a day. It would take about 20 min to half an hour. I feel better by learning extra things during the lockdown.

(P12-M-LMD)

“Since we didn’t go outside during the day, I went for a run early in the morning, then I read the relevant information about how we need to rely on our own immunity, that is, if we are healthy and our immunity is strong against viruses. You should be fine.”

(P14-M-CSS)

### 3.3. Stage Three: Post-Lockdown

#### 3.3.1. Stage Three—Part A: Caution

After 76 days, the Chinese Government lifted the lockdown. Older adults reported feeling cautiously optimistic about the evolution of the pandemic, and most chose not to go outdoors.

“I think it’s better not to go out after April. Since the epidemic has not been eliminated. Why are you going out? You are not going out to work, and you are not doing good for others, so why bother to others?”

(P9-F-QY)

“After April 8, I didn’t go out much. Unless it’s a necessity, we are over 65 years old, elderly people should go out less, and if the epidemic has not been eliminated, he will be held back in panic.”

(P3-F-WXJ)

#### 3.3.2. Stage Three—Part B: Trust and Gratitude 

The participants reflected upon their feelings and the connections of the government, nature, and society during the 76-day lockdown. Despite the temporary victory, some argued that this impactful experience provides important lessons pertaining to society’s relationship with nature. Others argued that the government should reflect on the pandemic and learn from this experience. Additionally, most older adults expressed gratitude to their government and country and emphasized their Chinese pride.

“Our government cares very much about its citizens. No matter where the disaster occurred, the country will continue to help and support it. Therefore, I feel very happy and proud to be born in such a country.”

(P20-F-CLS)

“We want to thank the comrades in the community for their help, and then we also want to thank the foreign aid from all over the country. The medical workers speak to our heart. If we want to change the course of the epidemic, it may require our involvement, right? Therefore, we cannot deny that we have benefited indirectly; we should not refrain from expressing our gratitude to these individuals from the depths of our hearts...”

(P18-M-WGB)

“Humans should learn to protect and respect nature. I think the two epidemics (the SARS and COVID-19) are too close. They have only been over ten years. I think from the public opinion, the country should reflect on what is the matter, the government should reflect on it, and what is the matter of the government? Every person should reflect on their experiences, because you can learn many things from them, these major events.”

(P15-F-CYJ)

## 4. Discussion

This study focused on examining the life experiences and behavioral and psychological adaptations of elderly individuals during the 76-day lockdown in Wuhan. Overall, our study revealed a noteworthy transition in the experiences, as they moved from initial fear and anxiety toward a sense of comfort and gratitude. This transition was facilitated by the implementation of various coping strategies, with social support playing a key role. Enhancing access to social support networks and connectivity can contribute to the overall well-being and resilience of older adults, not only during periods of lockdown, but also in their everyday lives. 

### 4.1. Transitioning from Fear to Adaptive Coping

Contrary to previous studies highlighting the vulnerability of older adults during the pandemic [32], our participants exhibited adaptive behavior, indicating their psychological coping and adaptability during this crisis [33]. The three coping strategies that emerged—staying busy, seeking social support, and maintaining a positive mindset—are consistent with proactive coping strategies aimed at minimizing the negative impact of social distance stressors [34]. 

It is not surprising that negative emotions, such as fear, anxiety, helplessness, and concern for family members, emerged during the pandemic. A number of studies have indicated that older adults specifically felt afraid of contracting the virus and facing mortality during the early phase of the pandemic [7,14,35]. The anxiety of individuals was further compounded by the lack of clear and concise guidelines during the lockdown period [30]. Moreover, the daily hardships and challenges faced by older adults necessitated the adoption of effective coping strategies [10]. In response to these adversities, various approaches were implemented to navigate these unprecedented circumstances and uphold mental well-being.

Our findings demonstrate that older adults’ self-coping styles included psychological and life adjustments, staying informed through reliable news sources, seeking support from family and friends, and engaging in online courses. These strategies were frequently observed in other research studies conducted globally during the pandemic [36,37]. Additionally, we came across several distinct approaches. For instance, numerous older individuals embarked on the journey of acquiring new social media skills and utilized them to foster emotional connections and forge solidarity with others. Although previous studies have highlighted the advantages of social media usage among older adults [38,39], the current study showed that older adults not only actively engaged with social media, but displayed a willingness to learn new tools. However, they were also consciously cautious about unreliable information on social media platforms. The findings suggest that healthcare professionals should effectively engage with older adults through social media platforms. They may also consider organizing webinars, online workshops, or Q&A sessions on social media platforms specifically for older adults [40]. These interactive sessions can provide them with an opportunity to learn about new tools and ask questions directly to healthcare experts. 

### 4.2. Strengthening Bonds: Reciprocal Social Supports 

There is no doubt that social support, such as the community’s provision of free food and medical supplies, as well as psychological comfort, significantly impact older adults’ emotional well-being [2]. Older adults often depend on social support systems, as their physical frailty necessitates it, and the mobilization of these resources is closely intertwined with the support they receive from diverse agents, such as the government, community, family, and volunteers [19,20,21,33]. 

The current study revealed that the participants not only received essential social support but also proactively assumed responsibilities in caring for their families, assisting neighbors, collaborating with the government, and actively extending social support to others. This finding aligns with the notion that older individuals play dual roles as recipients and providers of social support, actively engaging in social interactions [13,41]. Thomas [42] found that both receiving and giving social support can improve the well-being of older adults, while providing support for others plays a greater role. Similarly, Schulz and Sherwood [43] stated that by offering instrumental support to friends, relatives, and neighbors and providing emotional support to their spouse, the elderly can experience reduced depression, improved health, and decreased mortality. Nurain et al. [13] also confirmed that the roles of elders as both support providers and recipients were equally valued in overcoming various challenges during the pandemic. Other studies have shown that elderly people who give social support to others live longer [44] and improve life satisfaction [45,46]. 

Our findings have revealed that the concept of social support includes not only the exchange itself, but also the different levels of support offered by both the giver and the receiver. This understanding highlights the intricate dynamics involved in fostering effective social support networks. Recognizing the multifaceted nature of support is crucial in designing interventions and policies that cater to the diverse needs of individuals [47]. By emphasizing the importance of both giving and receiving support, we can enhance the quality of social interactions and promote collective well-being. Further research and practical applications should continue to explore and harness the transformative potential of social support in facilitating behavioral adaptation, and overall psychological well-being.

### 4.3. Caution, Trust, and Gratitude

We found that older adults expressed concerns after the lifting of the lockdown during the pandemic. The physical activities of older adults were still limited due to the precautionary measures taken to prevent infection [48]. While the reduced infection risk had its benefits, it should be acknowledged that it can also lead to physical and mental costs due to reduced activity and social contact [48,49,50]. In order to mitigate these effects, it is crucial to promote measures that prioritize both safety and well-being. Possible areas for improvement include enhanced testing, better assessment methods, increased social support, ensuring basic needs are met, and protecting homecare workers [51]. 

Although many participants supported the lockdown policy, there were exceptions such as allegations against medical staff and propaganda regarding the party’s and government’s political supremacy, which provoked anger, tension, and panic in some individuals. Other research also found that individuals’ distrust in the local government had a negative impact on their utilization of external resources, which in turn affected their psychological well-being during the pandemic [52,53,54]. Older adults may encounter greater challenges in dealing with the lockdown due to limited social support, financial resources, and self-regulation abilities [55]. Consequently, they may rely more heavily on the local government to address their needs and concerns during this period [56]. Healthcare professionals should further explore the trust in the government and its impact on the adaptive behaviors among older adults during the pandemic.

### 4.4. Limitations

The study encountered several limitations. Firstly, instead of using probability sampling, the data collection relied on convenience sampling, which was based on the personal connections of the authors. Thus, there is a potential for sampling bias in the study. It should also be noted that our participants have strong social connections within their communities. Consequently, it is possible that this sample may not adequately represent individuals who are currently dealing with serious living conditions. It is plausible that individuals who are facing more significant health challenges during the pandemic might have experienced heightened levels of distress. Despite efforts to recruit participants with diverse demographic characteristics such as gender, education, occupations, and living conditions, the study cannot guarantee generalizability. Secondly, the interview process was carried out as a single session for three different timelines and the participants were requested to recall their past experiences. They may have provided vague experiences or excluded important details. The ideal approach would be dividing the interview into three phases, addressing one timeline per phase. This would allow for the potential influence of the pandemic on participants’ perspectives to be reduced, as they may express different experiences during each timeline. However, due to concerns related to the ongoing pandemic and the heavy workload faced by the research assistants, we made the decision to conduct a single interview. Moreover, we conducted interviews through video calls or telephone. On one hand, it allowed us to capture experiences safely during the pandemic. However, it also created an equitable issue as those without internet or telephone access could not participate equitably and may have encountered additional challenges [57]. 

## 5. Conclusions

Understanding the life experiences of older adults and their adaptability and self-coping strategies is essential to effectively manage outbreaks. The participants in this study progressed through several stages of mental disturbance, adaptation, trust-building, reciprocal social support, and reflection. Notably, they exhibited an increase in gratitude, and engaged in reflection. These individuals directed their attention towards their future health and fostered deeper connections with nature, family, neighbors, and friends. Furthermore, they displayed an impressive ability to overcome early negative emotions by embracing and cultivating positive emotions. It is important to emphasize that older adults not only received social support but were also actively involved in providing support to their families, friends, communities, and others in need. This transformative journey highlights the immense capacity of older adults to adapt, find meaning, and flourish even in challenging circumstances [13,18,39,42,47]. Our research indicates that a combination of reciprocal social support received, and the use of self-coping strategies contributed significantly to overcoming negative emotions during the early stage of the pandemic and facilitating the transition back to normal life following the lifting of the lockdown. It is crucial for healthcare professionals to integrate these findings into their practice and encourage older adults to seek and nurture supportive relationships while also promoting the development of effective self-coping strategies [58,59]. This can greatly aid individuals in managing their emotional well-being during the pandemic period. 

## Figures and Tables

**Table 1:** Participants’ Demographic Characteristics.

Characteristics	Mean (SD) or N (%)
Gender	
Male	10 (50%)
Female	10 (50%)
Age, years (range = 65~85)	68.65 (4.30)
Education	
Junior high school	1 (5%)
Senior high school/Technical secondary school	9 (45%)
Undergraduate or higher	10 (50%)
Marital Status	
Single	1 (5%)
Married	18 (95%)
Widowed	1 (5%)
Work Status	
Yes, Consultant after retired	2 (10%)
No	18 (90%)
Living status	
Living alone	5 (25%)
Living with a caregiver	1 (5%)
Living with spouse	10 (50%)
Living with spouse and children and grandchildren	4 (20%)
Occupation before retirement	
Teachers (elementary and junior high school)	3 (15%)
Professors (college and above)	4 (20%)
Administrative workers	7 (35%)
Accountants	3 (15%)
Journalists	1 (5%)
Workers	1 (5%)
Nurses	1 (5%)

Note. SD = Standard Deviation; N = the total number of participants; % = the percentage based on 20 participants.

**Table 2:** Key Questions, Themes, and Related Codes During Each Stage of Lockdown.

Key Questions and Code	N (%)
**Stage 1: Early Lockdown** What emotions did you feel the most strongly during the first week of the lockdown?	
Nervousness	17 (85%)
Fear	14 (70%)
How did you cope with the situation during the first week of the lockdown in Wuhan?	
Searching for Support on Digital Media	10 (50%)
Searching for Answers on Digital Media	15 (75%)
Online shopping	12 (60%)
**Stage 2: Infection During Lockdown** How did you undergo a mental and psychological journey?	
From being unaccustomed to adaptation	11 (55%)
Can you share what has been most helpful or positive to you during the lockdown?	
Obtain Social Support	16 (80%)
Providing Support to Others	16 (80%)
Did your life undergo any changes during the lockdown?	
Adjusting a New Lifestyles	10 (50%)
Rebuilding Healthy Habits	11 (55%)
**Stage 3: Post-Lockdown**	
What changes have occurred in your mindset since the lockdown policy was lifted.	
Caution	11 (55%)
Optimism	14 (70%)
Did you undertake any psychological strategies to adapt to the emotional fluctuations?	
Trust	12 (60%)
Gratitude	14 (70%)

Note. N = the total number of participants under each code; % = the percentage based on 20 participants.

## Data Availability

The data presented in this study are available on request from the corresponding author. The data are not publicly available due to the confidentiality of the participants.

## Supplementary Materials

The following supporting information can be downloaded at: https://www.mdpi.com/article/10.3390/healthcare11222970/s1, Supplementary S1: Interview Questions; Supplementary S2: COREQ (Consolidated criteria for Reporting Qualitative research) Checklist.
