# Supplementary material for: Navigating Uncertainty: Experiences of Older Adults in Wuhan during the 76-Day COVID-19 Lockdown
Source: Healthcare (Basel). 2023 Nov 16;11(22):2970. doi: 10.3390/healthcare11222970 (PMC10671022; doi:10.3390/healthcare11222970)
Supplement: Supplementary file 1 [file healthcare-11-02970-s001.zip › healthcare-2639657-supplementary.pdf]

Supplementary S1. Interview Questions

First of all, thank you very much for agreeing to join us for this qualitative interview. As we all know, to deal with the impact of the novel coronavirus, on January 23, 2020, which coincided with the 29th day of the Chinese New Year, the Wuhan novel pneumonia prevention and control headquarters issued an order to suspend public transport, subway, ferry, and long-distance passenger transport in the city. Without special reasons, people were advised not to leave Wuhan, and the airport and railway station were temporarily closed. Since then, Wuhan entered a period of lockdown.

1. Generally speaking, during the nearly 100 days from the start of the lockdown to the end of the lockdown, please share the experience that has left the strongest impression on you. Please provide details of the time, place, person, event, and its impact on you.
   a) What emotions did you feel the most strongly during this time?
   b) Why do you think you felt these emotions?
   c) How did you undergo a mental and psychological journey? What changes did you experience and why?

2. During the first week of the epidemic in Wuhan, starting from January 23, people had limited knowledge about the virus, and the situation was unclear. When the sudden lockdown was announced:
   a) What were your predominant emotions?
   b) How did you cope with the situation?
   c) Who provided support to you during this time?
   d) Did you experience any unusual emotions or a sense of crisis? If so, what was your greatest concern?

3. Did your life undergo any changes during the lockdown?
   a) Did you experience any changes in your health?
   b) Did your daily activities and goals get affected? How?
   c) If yes, how did you feel about these changes? How did you adapt to them?
   d) How did you manage your daily life during the lockdown?
   e) Did these changes bring any positive aspects to your life?

4. Can you share what has been most helpful or positive to you during the pandemic?
   a) Why do you think these factors were helpful?
   b) Did you have any specific expectations or desires during this time?

5. Who were you with during the pandemic? Were you with family, friends, or others?
   a) Did they offer you any support? Did you find their support helpful?
   b) Did you have any interesting experiences or moments with them? Did you encourage each other to overcome difficulties together?

6. Were you influenced by news coverage during the pandemic?
   a) Did you follow news updates about the epidemic? What types of stories affected your mood?
   b) Did you increase the frequency of watching the news? What kind of news sources did you

mainly rely on? TV, radio, online news, or WeChat articles?

7. After Wuhan was unsealed on April 8, did you venture outside? How did you feel when you went out? Were you afraid of getting infected? If you didn't go out, how did you feel and why?

8. What changes have occurred in your mindset since Wuhan was unsealed? What is your greatest hope for the future?

9. In summary, having experienced this rare pandemic in a hundred years, how did you navigate through it? Did you undertake any psychological strategies to adapt to the emotional fluctuations?

10. What is the most important thing in your life right now?
   a) Do you have any goals for the future?
   b) Has something that was important to you in the past become less important now?
   c) Have things that used to be less important to you become more important now?
   d) Can you imagine a situation where life no longer makes sense?
   e) What is the most important thing to you if your health deteriorates?
   f) If you are unable to take care of yourself, what is the most important thing to you?

Supplementary question:
Is there anything I'm missing? Is there anything else you would like to share with me?

Supplementary S2. COREQ (Consolidated criteria for Reporting Qualitative research) Checklist

A checklist of items that should be included in reports of qualitative research. You must report the page number in your manuscript where you consider each of the items listed in this checklist. If you have not included this information, either revise your manuscript accordingly before submitting or note N/A.

Topic	Item No.	Guide Questions/Description	Reported on Page No.
**Domain 1: Research team and reflexivity**			
Personal characteristics			
Interviewer/facilitator	1	Which author/s conducted the interview or focus group?	3
Credentials	2	What were the researcher's credentials? E.g. PhD, MD	3
Occupation	3	What was their occupation at the time of the study?	1
Gender	4	Was the researcher male or female?	NA
Experience and training	5	What experience or training did the researcher have?	NA
Relationship with participants			
Relationship established	6	Was a relationship established prior to study commencement?	3
Participant knowledge of the interviewer	7	What did the participants know about the researcher? e.g. personal goals, reasons for doing the research	3
Interviewer characteristics	8	What characteristics were reported about the inter viewer/facilitator? e.g. Bias, assumptions, reasons and interests in the research topic	NA
**Domain 2: Study design**			
Theoretical framework			
Methodological orientation and Theory	9	What methodological orientation was stated to underpin the study? e.g. grounded theory, discourse analysis, ethnography, phenomenology, content analysis	2
Participant selection			
Sampling	10	How were participants selected? e.g. purposive, convenience, consecutive, snowball	3
Method of approach	11	How were participants approached? e.g. face-to-face, telephone, mail, email	3
Sample size	12	How many participants were in the study?	3
Non-participation	13	How many people refused to participate or dropped out? Reasons?	3
Setting			
Setting of data collection	14	Where was the data collected? e.g. home, clinic, workplace	3
Presence of non-participants	15	Was anyone else present besides the participants and researchers?	NA
Description of sample	16	What are the important characteristics of the sample? e.g. demographic data, date	4
Data collection			
Interview guide	17	Were questions, prompts, guides provided by the authors? Was it pilot tested?	3
Repeat interviews	18	Were repeat inter views carried out? If yes, how many?	NA
Audio/visual recording	19	Did the research use audio or visual recording to collect the data?	3
Field notes	20	Were field notes made during and/or after the inter view or focus group?	NA
Duration	21	What was the duration of the inter views or focus group?	3
Data saturation	22	Was data saturation discussed?	NA
Transcripts returned	23	Were transcripts returned to participants for comment and/or	NA

Topic	Item No.	Guide Questions/Description	Reported on Page No.
		correction?	
**Domain 3: analysis and findings**			
Data analysis			
Number of data coders	24	How many data coders coded the data?	3
Description of the coding tree	25	Did authors provide a description of the coding tree?	5
Derivation of themes	26	Were themes identified in advance or derived from the data?	NA
Software	27	What software, if applicable, was used to manage the data?	3
Participant checking	28	Did participants provide feedback on the findings?	NA
Reporting			
Quotations presented	29	Were participant quotations presented to illustrate the themes/findings? Was each quotation identified? e.g. participant number	6
Data and findings consistent	30	Was there consistency between the data presented and the findings?	NA
Clarity of major themes	31	Were major themes clearly presented in the findings?	10
Clarity of minor themes	32	Is there a description of diverse cases or discussion of minor themes?	11

Developed from: Tong A, Sainsbury P, Craig J. Consolidated criteria for reporting qualitative research (COREQ): a 32-item checklist for interviews and focus groups. *International Journal for Quality in Health Care*. 2007. Volume 19, Number 6: pp. 349 – 357

Once you have completed this checklist, please save a copy and upload it as part of your submission. DO NOT include this checklist as part of the main manuscript document. It must be uploaded as a separate file.
